# Supplementary material for: Computational imaging during video game playing shows dynamic synchronization of cortical and subcortical networks of emotions
Source: PLoS Biol. 2020 Nov 12;18(11):e3000900. doi: 10.1371/journal.pbio.3000900 (PMC7685507; doi:10.1371/journal.pbio.3000900)
Supplement: S1 Text — (DOCX) [file pbio.3000900.s001.docx]

SUPPLEMENTARY METHODS

*Computational modeling of feedback influences among components and evidence for their impact on synchronization*

In our modeling approach to compute transient synchronization between emotion components, feedback influences among the components were incorporated at the psycho-physiological level in our fitted time series model (ELSA). The ELSA model contained an internal dynamic system (=liquid state machine) whose degree of feedback recurrence among emotion components was controlled by two parameters, a scaling parameter lambda (λ), and an exponential decay parameter known as the leaking rate (α). Jointly, these two parameters will determine the eventual fading memory length of the synchronization model (e.g., 0 time steps, 60 time steps, etc.). Lambda scales the liquid weight matrix W such that its spectral radius can never exceed the lambda value. It is critical for the construction of the initial liquid weight matrix W and primarily ensures that W possesses the fading memory property without spuriously containing chaotic attractors. The impact of lambda on fading memory length is that larger values for lambda mean more lagged feedback, while smaller values mean less lagged feedback, with 0 implying virtually no feedback recurrence. Once the lambda value is set, it requires no further “optimization” during subsequent model estimation. By contrast, the leaking rate controls an amount of exponential decay on past states in the liquid state machine, and can dramatically alter the length of the fading memory such that it requires to be optimized with cross-validation during model estimation. The leaking rate takes values between 0 and 1, with 1 implying immediate decay of past states, and 0 implying (asymptotically) infinite decay of past states [Holzmann, G. and Hauser, H. (2010). Echo state networks with filter neurons and a delay&sum readout. Neural Netw. 23(2):244-56].

During model fitting, we set suitable values for lambda and the leaking rate by leave-one-run-outcross-validation procedure, using a forward search to set first the lambda value and second the leaking rate. This procedure arrived at an optimal feedback length of ca. 5000 time steps (λ = 0.8; α = 0.01), providing evidence that significant feedback was required for accurate modelling.

To bolster this finding further, these models were rerun for all participants (3 runs each × 20 random repetitions) with the amount of feedback reduced to its minimal possible number of 2 time steps (λ = 0; α = 1).^[[1]](#footnote-1)^ We compared the average observed-fitted correlations (*R*) of this analysis to the observed-fitted correlations obtained in the original analysis, and did this both for the training data and the test data. Results of this analysis are presented in Table S10, depicting average *R’*s for each emotion measure and each data set, as well as permutation *p*-values obtained from permutation paired *t*-tests.^[[2]](#footnote-2)^ Results indicate that the model fit for ApproachHead did not change significantly between feedback and no feedback for the training data, but did for the test data. For ApproachTail and EMG, the difference was not significant for either the training or test data, but these variables were difficult to model either way, with or without feedback. For the remaining five variables, however, there were highly significant decreases in average *R* when no feedback was allowed, both for the training and test data, suggesting that feedback was important for correct modelling, and hence for obtaining an accurate measure of synchronization for subsequent analyses.

In sum, our computational model based on psycho-physiological measurements during the video-game provides indirect support for the role of recurrent interactions and feedback between components during synchronization. In this framework, the driving forces of synchrony are precisely emerging from such reciprocal interactions between components, in line with dynamic system theories [Lewis, M. D. (2005). Bridging emotion theory and neurobiology through dynamic systems modeling. Behavioral and Brain Sciences, 28(2), 169–194], without having to assume any further external source imposing synchronization.

1. A feedback length of 2 steps is the minimum due to the model containing a symbolic “token” delay between the appraisal signals and the other component signals [↑](#footnote-ref-1)
2. Per test, 500 random sign flips were conducted to construct the empirical *t*-distribution [↑](#footnote-ref-2)
