# Supplementary material for: Computational imaging during video game playing shows dynamic synchronization of cortical and subcortical networks of emotions
Source: PLoS Biol. 2020 Nov 12;18(11):e3000900. doi: 10.1371/journal.pbio.3000900 (PMC7685507; doi:10.1371/journal.pbio.3000900)
Supplement: S1 Table — (DOCX) [file pbio.3000900.s002.docx]

SUPPLEMENTARY TABLES

**S1 Table A.** Related to Figure 4A. Effects of the Coping Potential Appraisal.

| *Brain Regions* | *MNI Coordinates (mm)* | | | *Z Score*  *(voxel)* | *p_FWE_-value*  *(voxel)* | *#Voxels in Cluster* | *p_FWE_-value*  *(cluster)* |
| --- | --- | --- | --- | --- | --- | --- | --- |
|  | x | y | z |  |  |  |  |
| ***Power _(good + neutral + bad)_ > No Power _(good + neutral + bad)_*** | | | | | | | |
| R Fusiform Gyrus | 34 | -54 | -20 | > 8 | < 0.001 | 2826 | < 0.001 |
| R Cerebellum (Lobule V) | 14 | -48 | -20 | 5.97 | < 0.001 |  |  |
| R Cerebellum (Lobule VI) | 6 | -66 | -12 | 5.67 | 0.001 |  |  |
| L Cerebellum (Lobule VI)/Fusiform | -32 | -58 | -20 | > 8 | < 0.001 | 1563 | < 0.001 |
| L Inferior Occipital Gyrus | -32 | -78 | -12 | 6.91 | < 0.001 |  |  |
| L Post-central Gyrus | -40 | 34 | 46 | 7.31 | < 0.001 | 603 | < 0.001 |
| L Pre-central Gyrus | -30 | -20 | 70 | 5.73 | < 0.001 |  |  |
| ***No Power _(good + neutral + bad)_ > Power _(good + neutral + bad)_*** | | | | | | | |
| R Anterior Cingulate Cortex (BA33) | 4 | 32 | -2 | 5.56 | 0.001 | 66 | < 0.001 |
| L Anterior Cingulate Cortex | -2 | 32 | -4 | 5.31 | 0.004 |  |  |
| L Anterior Insula (Ventral) | -38 | 10 | -12 | 5.37 | 0.003 | 45 | < 0.001 |

**S1 Table B.** Related to Figure 4B. Effects of Goal Conduciveness Appraisal.

| *Brain Regions* | *MNI Coordinates (mm)* | | | *Z Score*  *(voxel)* | *p_FWE_-value*  *(voxel)* | *#Voxels in Cluster* | *p_FWE_-value*  *(cluster)* |
| --- | --- | --- | --- | --- | --- | --- | --- |
|  | x | y | z |  |  |  |  |
| ***Good _(power + no power)_ > Bad _(power + no power)_*** | | | | | | | |
| L Rectal Gyrus | 0 | 38 | -20 | > 8 | < 0.001 | 2874 | < 0.001 |
| R Rectal Gyrus | 4 | 50 | -16 | 7.71 | < 0.001 |  |  |
| L Mid Orbital Gyrus | -4 | 48 | -10 | > 8 | < 0.001 |  |  |
| L Superior Medial Gyrus | -10 | 44 | 46 | 6.96 | < 0.001 |  |  |
| L Middle Frontal Gyrus | -32 | 24 | 52 | 7.11 | < 0.001 |  |  |
| L Superior Frontal Gyrus | -20 | 34 | 54 | 7.29 | < 0.001 |  |  |
| R Superior Frontal Gyrus | 16 | 42 | 50 | 7.80 | < 0.001 | 220 | < 0.001 |
| R Superior Medial Gyrus | 8 | 60 | 24 | 5.61 | 0.001 | 108 | < 0.001 |
| L Inferior Frontal Gyrus (p. Orbitalis) | -40 | 40 | -10 | 7.43 | < 0.001 | 428 | < 0.001 |
| L Middle Frontal Gyrus | -36 | 54 | 0 | 5.37 | 0.003 |  |  |
| R Inferior Frontal Gyrus (p. Orbitalis) | 38 | 38 | -12 | 6.75 | < 0.001 | 100 | < 0.001 |
| L Angular Gyrus | -42 | -68 | 40 | > 8 | < 0.001 | 1312 | < 0.001 |
| L Inferior Parietal Lobe | -34 | -72 | 48 | 7.76 | < 0.001 |  |  |
| L Superior Parietal Lobe | -30 | -60 | 58 | 6.12 | < 0.001 |  |  |
| R Angular Gyrus | 52 | -64 | 36 | 7.11 | < 0.001 | 263 | < 0.001 |
| L Posterior Cingulate Cortex | -2 | -40 | 30 | > 8 | < 0.001 | 1248 | < 0.001 |
| R Posterior Cingulate Cortex | 4 | -52 | 30 | 7.66 | < 0.001 |  |  |
| L Middle Temporal Gyrus | -58 | -38 | -12 | 7.67 | < 0.001 | 1095 | < 0.001 |
| L Middle Temporal Pole | -46 | 8 | -32 | 6.48 | < 0.001 |  |  |
| R Middle Temporal Gyrus | 58 | -38 | -10 | 5.05 | 0.013 | 21 | 0.001 |
| R Inferior Temporal Gyrus | 62 | -12 | -26 | 7.71 | < 0.001 | 579 | < 0.001 |
| R Medial Temporal Pole | 46 | 12 | -34 | 7.26 | < 0.001 |  |  |
| R Posterior Insula (dorsal) | 36 | -8 | 18 | 7.01 | < 0.001 | 513 | < 0.001 |
| R Post-central Gyrus | 66 | -12 | 18 | 6.93 | < 0.001 |  |  |
| R Post-central Gyrus (BA1) | 62 | -8 | 36 | 6.56 | < 0.001 |  |  |
| R Pre-central Gyrus | 60 | 4 | 34 | 5.66 | 0.001 |  |  |
| R Rolandic Operculum | 56 | -10 | 14 | 5.63 | 0.001 |  |  |
| R Pre-central Gyrus | 46 | -18 | 60 | 5.75 | < 0.001 | 18 | 0.002 |
| L Posterior Insula (dorsal) | -36 | -12 | 18 | 5.67 | 0.001 | 45 | < 0.001 |
| L Post-central Gyrus | -54 | -18 | 28 | 5.95 | < 0.001 | 72 | < 0.001 |
| L Post-central Gyrus | -54 | -10 | 14 | 5.44 | 0.002 | 58 | < 0.001 |
| L Superior Temporal Gyrus | -58 | -12 | 12 | 5.42 | 0.002 |  |  |
| L Hippocampus | -22 | -16 | -18 | 7.48 | < 0.001 | 317 | < 0.001 |
| R Parahippocampal Area | 22 | -14 | -18 | 6.82 | < 0.001 | 236 | < 0.001 |

**S1 Table B.** Related to Figure 4B. Effects of Goal Conduciveness Appraisal (*cont.*)

| *Brain Regions* | *MNI Coordinates (mm)* | | | *Z Score*  *(voxel)* | *p_FWE_-value*  *(voxel)* | *#Voxels in Cluster* | *p_FWE_-value*  *(cluster)* |
| --- | --- | --- | --- | --- | --- | --- | --- |
|  | x | y | z |  |  |  |  |
| ***Bad _(power + no power)_ > Good _(power + no power)_*** | | | | | | | |
| R Anterior Insula | 36 | 20 | 6 | > 8 | < 0.001 | 12155 | < 0.001 |
| R Superior Frontal Gyrus | 20 | 6 | 66 | > 8 | < 0.001 |  |  |
| R Precuneus | 8 | -56 | 58 | > 8 | < 0.001 |  |  |
| R Superior Parietal Lobe | 18 | -46 | 64 | > 8 | < 0.001 |  |  |
| R Middle Cingulate Cortex | 4 | 14 | 42 | > 8 | < 0.001 |  |  |
| L Middle Cingulate Cortex | -2 | 16 | 40 | > 8 | < 0.001 |  |  |
| R Fusiform Gyrus | 28 | -48 | -14 | > 8 | < 0.001 |  |  |
| R Calcarine Gyrus | 6 | -74 | 10 | > 8 | < 0.001 |  |  |
| L Calcarine Gyrus | -12 | -70 | 8 | > 8 | < 0.001 |  |  |
| L Anterior Insula | -32 | 18 | 8 | > 8 | < 0.001 | 724 | < 0.001 |
| R Inferior Frontal Gyrus | 46 | -62 | -14 | 5.92 | < 0.001 | 135 | < 0.001 |
| L Posterior-Medial Frontal Gyrus | -10 | -8 | 74 | 5.48 | 0.002 | 95 | < 0.001 |
| R Middle Frontal Gyrus | 36 | 46 | 18 | 7.25 | < 0.001 | 872 | < 0.001 |
| L Superior Parietal Lobe | -16 | -46 | 64 | 7.61 | < 0.001 | 691 | < 0.001 |
| L Pre-central Gyrus | -26 | -28 | 54 | 6.34 | < 0.001 |  |  |
| L Middle Cingulate Cortex/BA5 | -14 | -32 | 44 | 6.17 | < 0.001 |  |  |
| R Supramarginal Gyrus | 58 | -34 | 34 | 7.56 | < 0.001 | 1125 | < 0.001 |
| L Supramarginal Gyrus | -56 | -34 | 26 | 5.56 | 0.001 | 91 | < 0.001 |
| R Middle Temporal Gyrus | 42 | -70 | 16 | > 8 | < 0.001 | 592 | < 0.001 |
| L Superior Occipital Gyrus | -14 | -80 | 40 | 5.21 | 0.006 | 27 | 0.001 |
| R Thalamus (Anterior) | 6 | -18 | 12 | > 8 | < 0.001 | 447 | < 0.001 |
| R Thalamus (Ventrolateral) | 6 | -20 | 0 | 7.68 | < 0.001 |  |  |
| L Thalamus (Anterior) | -12 | -20 | 16 | 5.51 | 0.001 | 18 | 0.002 |
| R Caudate Nucleus | 12 | 0 | 14 | 6.77 | < 0.001 | 34 | < 0.001 |
| L Caudate Nucleus | -10 | 2 | 12 | 5.41 | 0.002 | 16 | 0.002 |

**S1 Table C.** Related to Figure 4C. Interaction Effects between Goal Conduciveness and Coping Potential Appraisals

| *Brain Regions* | *MNI Coordinates (mm)* | | | *Z Score*  *(voxel)* | *p_FWE_-value*  *(voxel)* | *#Voxels in Cluster* | *p_FWE_-value*  *(cluster)* |
| --- | --- | --- | --- | --- | --- | --- | --- |
|  | x | y | z |  |  |  |  |
| ***Bad _(no power > power)_ > Good _(no power > power)_*** | | | | | | | |
| L Cerebellum (Crus 1) | -40 | -52 | -32 | 6.77 | < 0.001 | 442 | < 0.001 |
| L Cerebellum (Lobule VI) | -28 | -60 | -26 | 6.35 | < 0.001 |  |  |
| Cerebellar Vermis | 0 | -58 | -34 | 6.88 | < 0.001 | 189 | < 0.001 |
| R Cerebellum (Lobule VI) | 32 | -52 | -30 | 5.50 | 0.001 | 77 | < 0.001 |
| R Anterior Insula | 30 | 20 | -10 | 5.90 | < 0.001 | 213 | < 0.001 |
| R Inferior Frontal Gyrus | 44 | 18 | 4 | 5.29 | 0.004 |  |  |
| L Anterior Insula | -28 | 16 | -10 | 6.13 | < 0.001 | 44 | < 0.001 |
| L Posterior-Medial Frontal Gyrus | -8 | -2 | 72 | 5.52 | 0.001 | 126 | < 0.001 |
| R Posterior-Medial Frontal Gyrus | 8 | -2 | 68 | 5.93 | < 0.001 | 98 | < 0.001 |
| R Middle Cingulate Cortex | 4 | 22 | 30 | 5.59 | 0.001 | 41 | < 0.001 |
| L Middle Cingulate Cortex | -2 | 8 | 36 | 5.20 | 0.006 | 39 | < 0.001 |
| R Superior Parietal Lobe | 12 | -54 | 62 | 5.33 | 0.003 | 39 | < 0.001 |
| L Superior Parietal Lobe | -16 | -54 | 62 | 5.12 | 0.009 | 13 | 0.004 |
| Periaqueductal Gray | 2 | -28 | -4 | 6.15 | < 0.001 | 59 | < 0.001 |
| R Thalamus (Dorsomedial) | 6 | -18 | 14 | 5.81 | < 0.001 | 24 | 0.001 |
| ***Good _(no power > power)_ > Bad _(no power > power)_*** | | | | | | | |
| L Inferior Frontal Gyrus (p. Triang.) | -40 | 30 | 14 | 6.66 | < 0.001 | 378 | < 0.001 |
| L Inferior Frontal Gyrus (p. Orbitalis) | -40 | 42 | -12 | 5.66 | 0.001 | 129 | < 0.001 |
| R Inferior Frontal Gyrus (p. Triang.) | 46 | 38 | 10 | 5.45 | 0.002 | 83 | < 0.001 |
| L Middle Frontal Gyrus | -46 | 12 | 36 | 5.49 | 0.001 | 95 | < 0.001 |
| L Rolandic Operculum | -40 | -14 | 20 | 6.28 | < 0.001 | 92 | < 0.001 |
| R Rolandic Operculum | 36 | -12 | 18 | 6.70 | < 0.001 | 84 | < 0.001 |
| L Superior Parietal Lobe | -30 | -64 | 48 | 5.72 | < 0.001 | 128 | < 0.001 |
| R Amygdala | 26 | 0 | -24 | 5.40 | 0.002 | 28 | 0.001 |
| R Hippocampus | 30 | -6 | -22 | 4.98 | 0.017 |  |  |
| L Lingual Gyrus | -16 | -52 | 0 | 5.45 | 0.002 | 30 | 0.001 |
| R Lingual Gyrus | 18 | -46 | 0 | 5.45 | 0.002 | 29 | 0.001 |

**S1 Table D.** Related to Figure 5A. Motivation Network

| *Brain Regions* | *MNI Coordinates (mm)* | | | *Z Score*  *(voxel)* | *p_FWE_-value*  *(voxel)* | *#Voxels in Cluster* | *p_FWE_-value*  *(cluster)* |
| --- | --- | --- | --- | --- | --- | --- | --- |
|  | x | y | z |  |  |  |  |
| ***Motivation (+)*** | | | | | | | |
| R Superior Temporal Gyrus | 60 | -22 | 8 | 7.31 | < 0.001 | 2314 | < 0.001 |
| R Heschl’s Gyrus | 40 | -22 | 10 | 5.64 | 0.001 |  |  |
| R Posterior Insula | 36 | -20 | 14 | 5.62 | 0.001 |  |  |
| L Middle Temporal Gyrus | -62 | -36 | 6 | 5.85 | < 0.001 | 1989 | < 0.001 |
| L Superior Temporal Gyrus | -58 | -28 | 6 | 5.85 | < 0.001 |  |  |
| L Heschl’s Gyrus | -36 | -26 | 12 | 5.33 | 0.004 |  |  |
| L Inferior Parietal Lobe | -28 | -76 | 42 | 5.30 | 0.005 | 831 | < 0.001 |
| L Superior Parietal Lobe | -22 | -64 | 60 | 5.21 | 0.007 |  |  |
| R Superior Parietal Lobe | 26 | -60 | 62 | 4.66 | 0.086 | 88 | 0.041 |
| L Anterior Cingulate Cortex | -8 | 40 | -6 | 5.89 | <0.001 | 799 | < 0.001 |
| L Mid Orbital Gyrus | -4 | 48 | -10 | 5.39 | 0.003 |  |  |
| R Superior Medial Gyrus | 14 | 64 | 18 | 4.28 | > 0.05 | 107 | 0.018 |
| L Inferior Frontal Gyrus (p. Orbitalis) | -28 | 32 | -12 | 4.46 | > 0.05 | 93 | 0.033 |
| L Middle Cingulate Cortex | -2 | -32 | 34 | 5.00 | 0.020 | 119 | 0.011 |
| R Pre-central Gyrus | 14 | -28 | 74 | 4.85 | 0.038 | 166 | 0.002 |
| L Paracentral Gyrus (BA4a) | -6 | -30 | 72 | 3.81 | > 0.05 | 81 | 0.057 |
| R Lingual Gyrus | 12 | -76 | -8 | 5.65 | 0.001 | 470 | < 0.001 |
| L Lingual Gyrus | -12 | -76 | -4 | 5.36 | 0.003 |  |  |
| L Precuneus | -6 | -56 | 18 | 5.83 | < 0.001 | 441 | < 0.001 |
| L Posterior Cingulate Cortex | -12 | -50 | 30 | 5.10 | 0.013 |  |  |
| L Middle Occipital Gyrus | -42 | -72 | 16 | 4.39 | > 0.05 | 190 | 0.001 |
| ***Motivation (-)*** | | | | | | | |
| R Middle Cingulate Cortex | 6 | 12 | 44 | 6.77 | < 0.001 | 1716 | < 0.001 |
| R Posterior-Medial Frontal Gyrus | 16 | 0 | 70 | 5.87 | < 0.001 |  |  |
| R Superior Frontal Gyrus | 20 | 6 | 62 | 5.58 | 0.001 |  |  |
| L Middle Cingulate Cortex | -6 | 22 | 32 | 4.73 | 0.063 |  |  |
| R Middle Cingulate Cortex | 14 | -26 | 42 | 4.57 | > 0.05 | 145 | 0.004 |
| R Middle Frontal Gyrus | 30 | 48 | 18 | 5.02 | 0.018 | 308 | < 0.001 |
| R Pre-central Gyrus | 42 | 4 | 46 | 4.26 | > 0.05 | 108 | 0.017 |
| L Superior Frontal Gyrus | -16 | 0 | 72 | 4.90 | 0.031 | 178 | 0.001 |
| R Inferior Frontal Gyrus | 48 | 18 | -2 | 6.73 | < 0.001 | 1036 | < 0.001 |
| R Anterior Insula | 34 | 24 | -2 | 6.56 | < 0.001 |  |  |
| L Anterior Insula | -32 | 18 | 6 | 4.86 | 0.037 | 240 | < 0.001 |
| R Supramarginal Gyrus | 56 | -28 | 32 | 5.07 | 0.014 | 612 | < 0.001 |
| R Post-central Gyrus | 52 | -24 | 42 | 4.83 | 0.042 |  |  |
| L Inferior Parietal Lobe | -52 | -32 | 46 | 4.60 | > 0.05 | 185 | 0.001 |
| L Superior Parietal Lobe | -20 | -44 | 64 | 4.94 | 0.026 | 113 | 0.014 |
| R Precuneus | 10 | -68 | 48 | 4.87 | 0.036 | 369 | < 0.001 |
| L Cerebellum (VI) | -34 | -72 | -22 | 6.66 | < 0.001 | 1490 | < 0.001 |
| L Cerebellum (Crus 1) | -40 | -64 | -24 | 6.45 | < 0.001 |  |  |
| Cerebellar Vermis | 0 | -62 | -10 | 5.89 | < 0.001 | 422 | < 0.001 |
| R Thalamus (Anterior) | 8 | -10 | 10 | 5.30 | 0.023 | 168 | 0.002 |
| R Inferior Occipital Gyrus | 38 | -80 | -12 | 6.80 | < 0.001 | 1109 | < 0.001 |
| R Cuneus | 14 | -98 | 10 | 5.97 | < 0.001 |  |  |
| R Middle Occipital Gyrus | 34 | -86 | 10 | 5.82 | < 0.001 |  |  |
| L Middle Occipital Gyrus | -10 | -102 | 0 | 7.60 | < 0.001 | 316 | < 0.001 |

**S1 Table E.** Related to Figure 5B. Expression Network

| *Brain Regions* | *MNI Coordinates (mm)* | | | *Z Score*  *(voxel)* | *p_FWE_-value*  *(voxel)* | *#Voxels in Cluster* | *p_FWE_-value*  *(cluster)* |
| --- | --- | --- | --- | --- | --- | --- | --- |
|  | x | y | z |  |  |  |  |
| ***Expression (+)*** | | | | | | | |
| L Post-central Gyrus | -60 | -2 | 20 | 4.63 | > 0.05 | 614 | < 0.001 |
| L Post-central Gyrus (Area 4p) | -42 | -18 | 38 | 4.44 | > 0.05 |  |  |
| R Rolandic Operculum | 60 | -6 | 14 | 5.52 | 0.002 | 188 | < 0.001 |
| R Post-central Gyrus | 60 | -10 | 24 | 3.70 | > 0.05 |  |  |
| R Pre-central Gyrus | 58 | -2 | 40 | 4 | > 0.05 | 91 | 0.014 |
| R Post-central Gyrus | 60 | -12 | 42 | 3.53 | > 0.05 |  |  |
| L Cuneus | -2 | -82 | 36 | 4.53 | > 0.05 | 91 | 0.014 |
| R Cuneus | 6 | -80 | 42 | 3.22 | > 0.05 |  |  |
| R Cerebellum (VI) | 20 | -60 | -18 | 4.52 | > 0.05 | 114 | 0.004 |
| ***Expression (-)*** | | | | | | | |
| L Middle Temporal Gyrus | -62 | -14 | -4 | 4.14 | > 0.05 | 121 | 0.003 |
| R Middle Temporal Gyrus | 66 | -30 | 0 | 4.07 | > 0.05 | 84 | 0.020 |
| R Superior Temporal Gyrus | 68 | -20 | 0 | 3.76 | > 0.05 |  |  |
| R Putamen | 18 | 10 | -6 | 4.89 | 0.053 | 73 | 0.038 |
| R Caudate Nucleus | 12 | 14 | -4 | 4.06 | > 0.05 |  |  |

**S1 Table F.** Related to Figure 5C. Physiology Network

| *Brain Regions* | *MNI Coordinates (mm)* | | | *Z Score*  *(voxel)* | *p_FWE_-value*  *(voxel)* | *#Voxels in Cluster* | *p_FWE_-value*  *(cluster)* |
| --- | --- | --- | --- | --- | --- | --- | --- |
|  | x | y | z |  |  |  |  |
| ***Physiology (+)*** | | | | | | | |
| L Cerebellum | -6 | -82 | -18 | 7.64 | < 0.001 | 3266 | < 0.001 |
| Cerebellar Vermis | 0 | -64 | -32 | 6.74 | < 0.001 |  |  |
| R Cerebellum | 20 | -44 | -22 | 5.20 | 0.009 |  |  |
| R Middle Frontal Gyrus | 36 | 38 | 20 | 5.38 | 0.004 | 1740 | < 0.001 |
| R Superior Frontal Gyrus | 20 | 0 | 72 | 5.03 | 0.021 |  |  |
| R Anterior Cingulate Cortex | 6 | 44 | 4 | 5.26 | 0.007 | 933 | < 0.001 |
| R Superior Medial Gyrus | 12 | 48 | 38 | 4.93 | 0.032 |  |  |
| R Superior Frontal Gyrus | 24 | 48 | 38 | 4.72 | 0.077 |  |  |
| R Middle Cingulate Cortex | 10 | 42 | 30 | 4.72 | 0.079 |  |  |
| R Middle Cingulate Cortex | 2 | -16 | 38 | 3.94 | > 0.05 | 99 | 0.019 |
| R Supramarginal Gyrus | 52 | -34 | 36 | 5.05 | 0.019 | 469 | < 0.001 |
| L Post-central Gyrus | -38 | -30 | 46 | 4.07 | > 0.05 | 112 | 0.010 |
| R Precuneus | 6 | -60 | 64 | 4.86 | 0.044 | 102 | 0.016 |
| R Middle Occipital Gyrus | 36 | -76 | 10 | 4.52 | > 0.05 | 101 | 0.017 |
| ***Physiology (-)*** | | | | | | | |
| R Pre-central Gyrus | 46 | -18 | 58 | 6.09 | < 0.001 | 1521 | < 0.001 |
| R Posterior-Medial Frontal Gyrus | 4 | -24 | 56 | 5.84 | < 0.001 |  |  |
| L Posterior-Medial Frontal Gyrus | -2 | -14 | 60 | 5.67 | 0.001 |  |  |
| L Mid Orbital Gyrus | -4 | 42 | -14 | 5.32 | 0.005 | 261 | < 0.001 |
| L Posterior Cingulate Cortex | -2 | -50 | 20 | 4.08 | > 0.05 | 94 | 0.023 |
| R Rolandic Operculum | 38 | -16 | 18 | 4.88 | 0.039 | 239 | < 0.001 |
| R Posterior Insula | 34 | -20 | 10 | 3.97 | > 0.05 |  |  |
| L Inferior Parietal Lobe | -30 | -72 | 50 | 4.76 | 0.067 | 532 | < 0.001 |
| R Angular Gyrus | 50 | -66 | 38 | 4.56 | > 0.05 | 127 | 0.005 |
| L Middle Temporal Gyrus | -56 | -40 | 6 | 4.85 | 0.046 | 446 | < 0.001 |
| R Middle Temporal Gyrus | 56 | 0 | -26 | 4.84 | 0.048 | 281 | < 0.001 |
| R Superior Temporal Gyrus | 54 | -10 | 2 | 4.93 | 0.032 | 128 | 0.005 |
| L Temporal Pole | -38 | 18 | -30 | 4.37 | > 0.05 | 93 | 0.025 |
| R Inferior Occipital Cortex | 50 | -76 | -2 | 6.06 | < 0.001 | 159 | 0.001 |
| R Middle Occipital Cortex | 48 | -80 | 0 | 5.66 | 0.001 |  |  |
| R Amygdala | 24 | 2 | -12 | 6.83 | < 0.001 | 1521 | < 0.001 |
| L Putamen | -24 | 2 | -10 | 5.96 | < 0.001 |  |  |
| R Putamen | 24 | 6 | 6 | 5.52 | 0.002 |  |  |
| R Caudate Nucleus | 14 | 14 | 2 | 5.96 | < 0.001 |  |  |
| L Caudate Nucleus | -12 | 10 | 4 | 5.90 | < 0.001 |  |  |
| L Thalamus (Anterior) | -6 | -18 | 6 | 5.28 | 0.006 | 269 | < 0.001 |
| R Thalamus (Anterior) | 8 | -12 | 10 | 4.82 | 0.052 |  |  |

**S1 Table G.** Related to Figure 6. Center of Mass (CoM) Coordinates of Intersection Clusters between the two Synchronization Indices.

| *Brain Regions* | *CoM - MNI Coordinates (mm)* | | | *#Voxels in Cluster* |
| --- | --- | --- | --- | --- |
|  | x | y | z |  |
| R Primary Visual Cortex | 16 | -93 | 2 | 51 |
| L Primary Visual Cortex | -4 | -84 | 15 | 132 |
| L Visual Cortex | -11 | -66 | -4 | 40 |
| R Auditory Cortex | 57 | 4 | -4 | 43 |
| R Primary Motor Cortex | 28 | -35 | 66 | 10 |
| R Middle Temporal Gyrus | 62 | -19 | -10 | 337 |
| L Middle Temporal Gyrus | -58 | -24 | -11 | 552 |
| L Superior Temporal Gyrus | -61 | -32 | 13 | 29 |
| R Medial Temporal Pole | 43 | 11 | -35 | 49 |
| L Medial Temporal Pole | -43 | 12 | -33 | 112 |
| R Posterior Insula | 41 | -17 | 0 | 31 |
| R Operculum | 43 | -21 | 19 | 44 |
| L Operculum | -44 | -14 | -3 | 13 |
| L Inferior Parietal Lobe | -52 | -56 | 26 | 443 |
| R Superior Parietal Lobe (BA5) | 7 | -36 | 52 | 22 |
| L Posterior Cingulate Cortex/Precuneus | -4 | -51 | 35 | 215 |
| L Superior Medial Frontal Gyrus | --8 | 50 | 30 | 307 |
| L Superior Middle Frontal Gyrus | -23 | 28 | 48 | 167 |
| R Fusiform Gyrus/Cerebellum | 35 | -70 | -22 | 10 |
| R Putamen (Ventral) | 25 | 6 | -9 | 30 |
| R Putamen (Dorsal) | 24 | 6 | 4 | 14 |
| L Putamen | -19 | 10 | -10 | 7 |

*Given that these results were obtained through the intersection of the activation clusters obtained from the two synchronization approaches, the clusters are represented here in terms of center of mass (CoM), rather than through the standard activation peaks. Note that the CoM only lies within the cluster if this consists of a convex set of voxels, which is not necessarily the case for all the clusters. The CoM coordinates thus provides an approximate location of the clusters, while its exact coordinates should not be taken in an absolute way.*

**S1 Table H.** Related to Discussion. Game scores per condition averaged across participants (± Standard Deviation).

|  | *No Power* | | | *Power* | | |
| --- | --- | --- | --- | --- | --- | --- |
|  | *Good* | *Neutral* | *Bad* | *Good* | *Neutral* | *Bad* |
| *Scores* | 126.27 ± 12.48 | 74.84 ± 4.89 | 5.72 ± 4.96 | 764.97 ± 136.15 | 75.28 ± 4.64 | 29.05 ± 9.78 |

**S1 Table I.** Related to Methods - Participants. Behavioral responses for the core module of the Game Experience Questionnaire ^6,11^.

| *Game Facet* | *Mean* | *Standard Deviation* | *t-value* | *p-value* |
| --- | --- | --- | --- | --- |
| *competence* | 3.57 | 0.66 | 4.4 | <0.001 |
| *immersion* | 2.82 | 0.82 | -1.11 | 0.14 |
| *flow* | 3.58 | 0.94 | 3.17 | 0.002 |
| *tension* | 1.95 | 0.87 | -6.17 | <0.001 |
| *challenge* | 2.65 | 0.77 | -2.29 | 0.018 |
| *negative Affect* | 1.85 | 0.70 | -8.36 | <0.001 |
| *positive Affect* | 3.65 | 0.71 | 4.61 | <0.001 |

*Each of the game facets was rated using 5-point Likert scales (Not at all (=1), Slightly (=2), Moderately (=3), Fairly (=4), Extremely (=5)). Facet scores were compared with the intermediate value (‘moderately’) using t-tests and permutation based on sign flipping matrices to determine statistical significance (number of permutation equaled 5000). Average values higher than three indicate that the facet was rated as been felt more than moderately during the game, while values lower than three indicate that the facet was weakly felt.*

**S1 Table J**. Comparison of average fit statistics (observed-fitted correlation; *R*) when feedback versus when no feedback (no feedb.) is allowed in the synchronization model (feedb.). Comparisons were conducted by permutation paired *t*-tests. p ∙ < 0.1, * < 0.05, ** < 0.01, *** < 0.001.

|  | **Average training fit (*R*)** | | |  | **Average test fit (*R*)** | | |  |
| --- | --- | --- | --- | --- | --- | --- | --- | --- |
| **Variable** | **Feedb.** | **No feedb.** | **Perm. *p*** |  | **Feedb.** | **No feedb.** | **Perm. *p*** |  |
| ApproachHead (Mot) | 0.183 | 0.170 | 0.150 |  | 0.171 | 0.154 | 0.012 | * |
| ApproachTail (Mot) | 0.014 | 0.000 | 0.164 |  | 0.010 | 0.000 | 0.142 |  |
| EatenCoins (Mot) | 0.299 | 0.166 | < 0.001 | *** | 0.266 | 0.136 | < 0.001 | *** |
| Countdown (Mot) | 0.297 | 0.103 | < 0.001 | *** | 0.260 | 0.094 | < 0.001 | *** |
| Heart Rate (Phy) | 0.242 | 0.030 | < 0.001 | *** | 0.147 | 0.018 | < 0.001 | *** |
| RR (Phy) | 0.193 | 0.044 | < 0.001 | *** | 0.148 | 0.031 | < 0.001 | *** |
| EDA (Phy) | 0.219 | 0.008 | < 0.001 | *** | 0.160 | 0.003 | < 0.001 | *** |
| EMG (Exp) | 0.024 | 0.000 | 0.138 |  | 0.011 | 0.000 | 0.166 |  |
