# Supplementary material for: Computational imaging during video game playing shows dynamic synchronization of cortical and subcortical networks of emotions
Source: PLoS Biol. 2020 Nov 12;18(11):e3000900. doi: 10.1371/journal.pbio.3000900 (PMC7685507; doi:10.1371/journal.pbio.3000900)
Supplement: S1 Fig — (DOCX) [file pbio.3000900.s003.docx]

SUPPLEMENTARY FIGURES

**
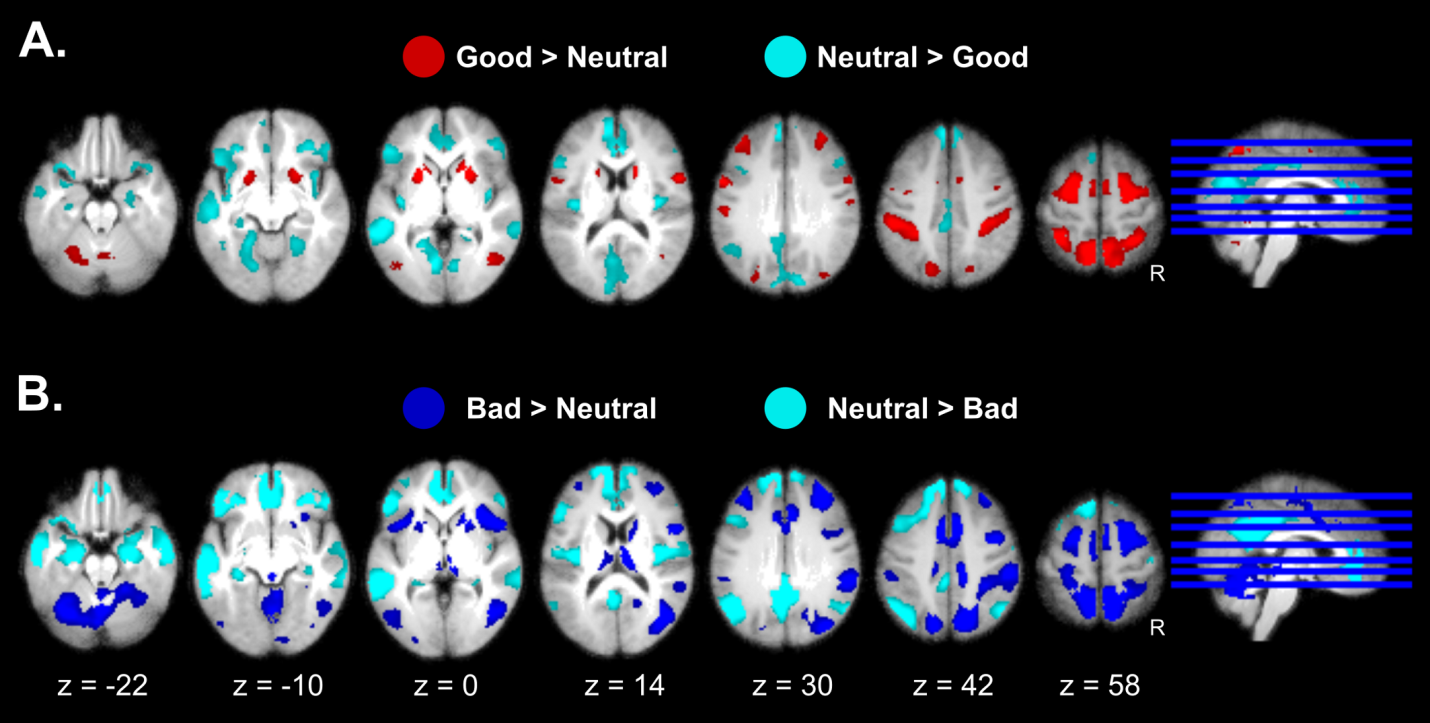
S1 Fig A**. (**Related to Fig 4) Effects of Goal Conduciveness are shown separately for the (A) good monster conditions (B) bad monster conditions**. Effects are presented on axial slices of a mean brain image created by averaging the participants' normalized structural images, with a statistical threshold of *p_FWE_* < 0.05. Individual beta maps used for this figure are available on Neurovault at https://identifiers.org/neurovault.collection: 8740.


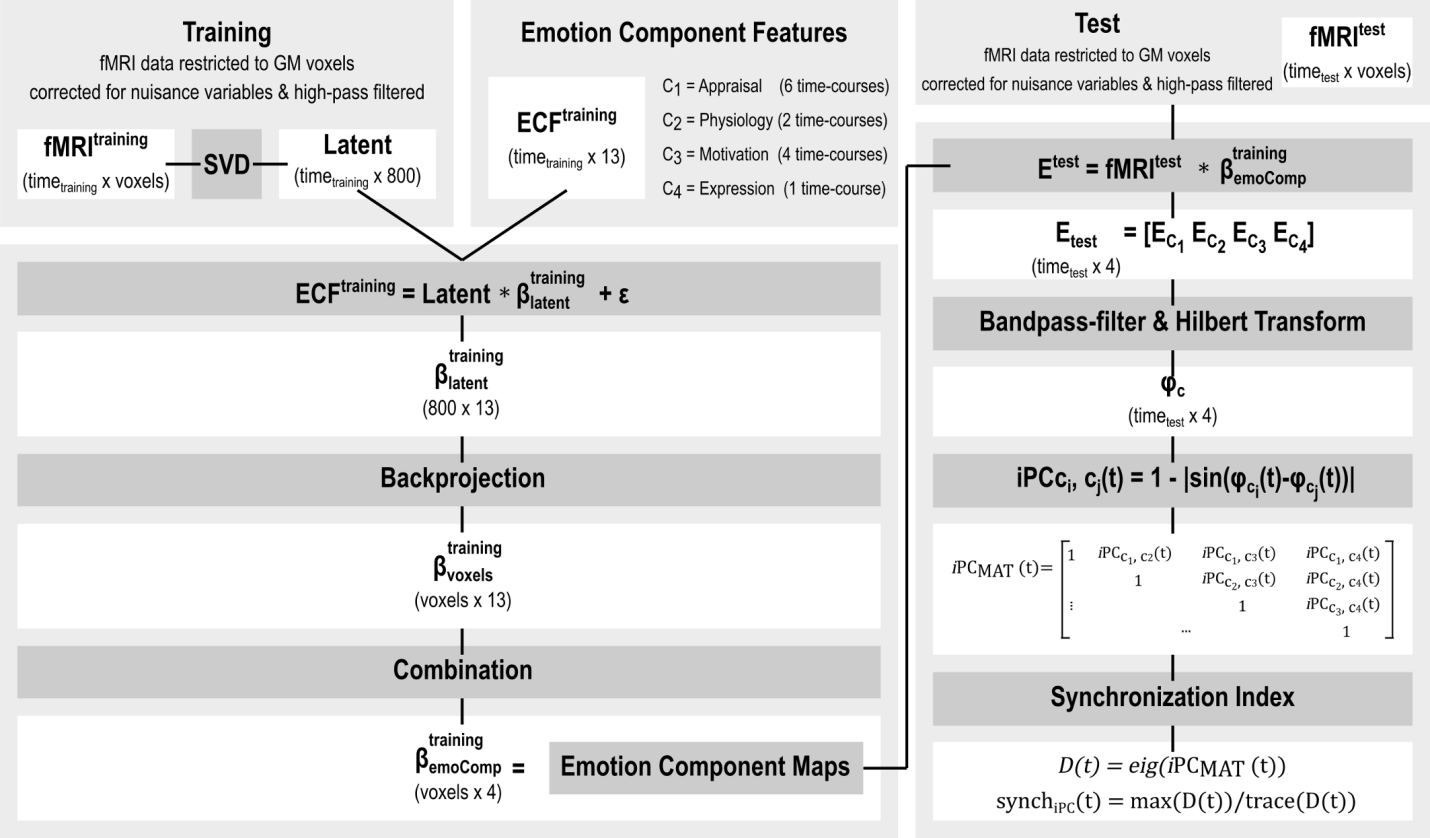


**S1 Fig B**. **(Related to Fig 6) Detailed overview of the algorithmic steps taken in the derivation of the Brain-based Synchronization Index.** (left) Emotion Component Maps were derived by predicting Emotion Component Features from fMRI data in a SVD regression using the training data set and combining the ensuing feature maps of each Emotion Component. (right) Representative Emotion Component time-courses were derived by calculating the time-point by time-point scalar product between the Emotion Component Maps obtained from the training data set and the BOLD time-course of the test run. The Hilbert Transform was applied to the bandpass filtered representative Emotion Component time-courses to derive the instantaneous phase of these signals, which was used to calculate the instantaneous phase coherence matrix at each time-point, consisting of the pair-wise instantaneous phase coherence between each pair of Emotion Components. The instantaneous phase coherence matrix at each time-point was used to calculate a temporal multivariate synchronization index, by calculating the maximum eigenvalue of this matrix normalized by the sum of all eigenvalues.


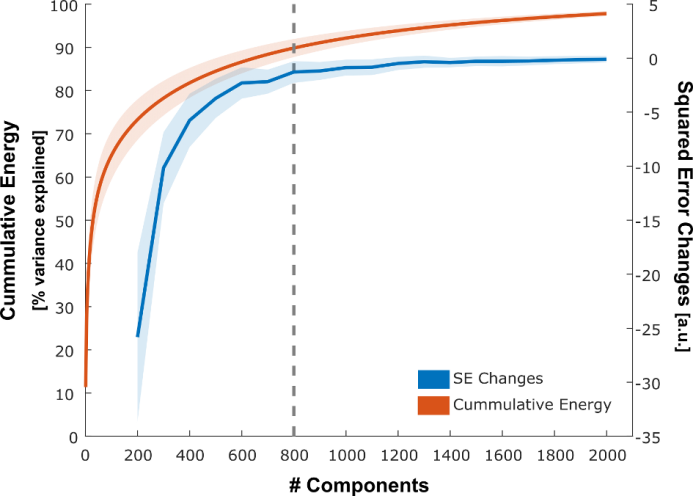


**S1 Fig C. (Related to Fig 6 and Fig SB)** **Criteria for chosen number of SVD components**. The number of leading components used for the latent data set was chosen based on the cumulative energy and on the change in squared error calculated between the predicted (obtained from the training data set) and the empirical (test run) time-courses. Data used in this figure can be found in S3 Data.

**
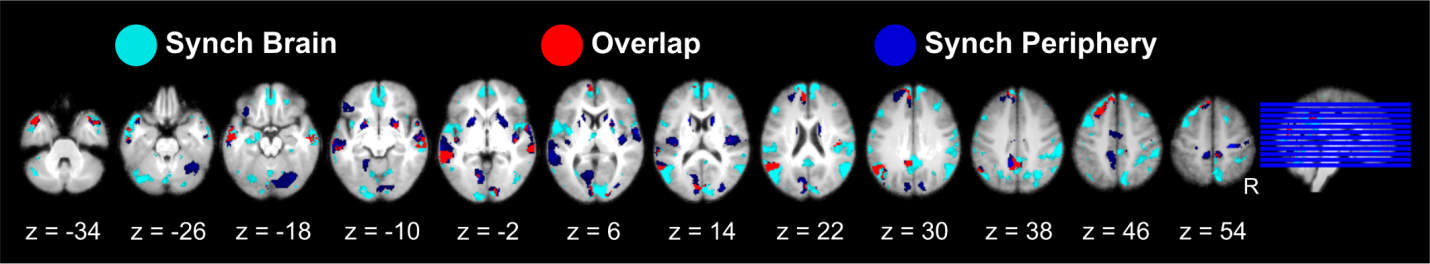
**

**S1 Fig D. (Related to Fig 7) Brain activation patterns associated with synchronization between emotion components**, as estimated by a “central” brain-based synchronization index derived from the activity time-course of component brain networks (cyan) and a “periphery” model-based synchronization index determined by independent computational modelling of peripheral and behavioral measures (blue), as well as their overlap (red). Effects are presented on axial slices of a mean image created by averaging the participants' normalized structural images. Individual beta maps used for this figure are available on Neurovault at https://identifiers.org/neurovault.collection: 8740.


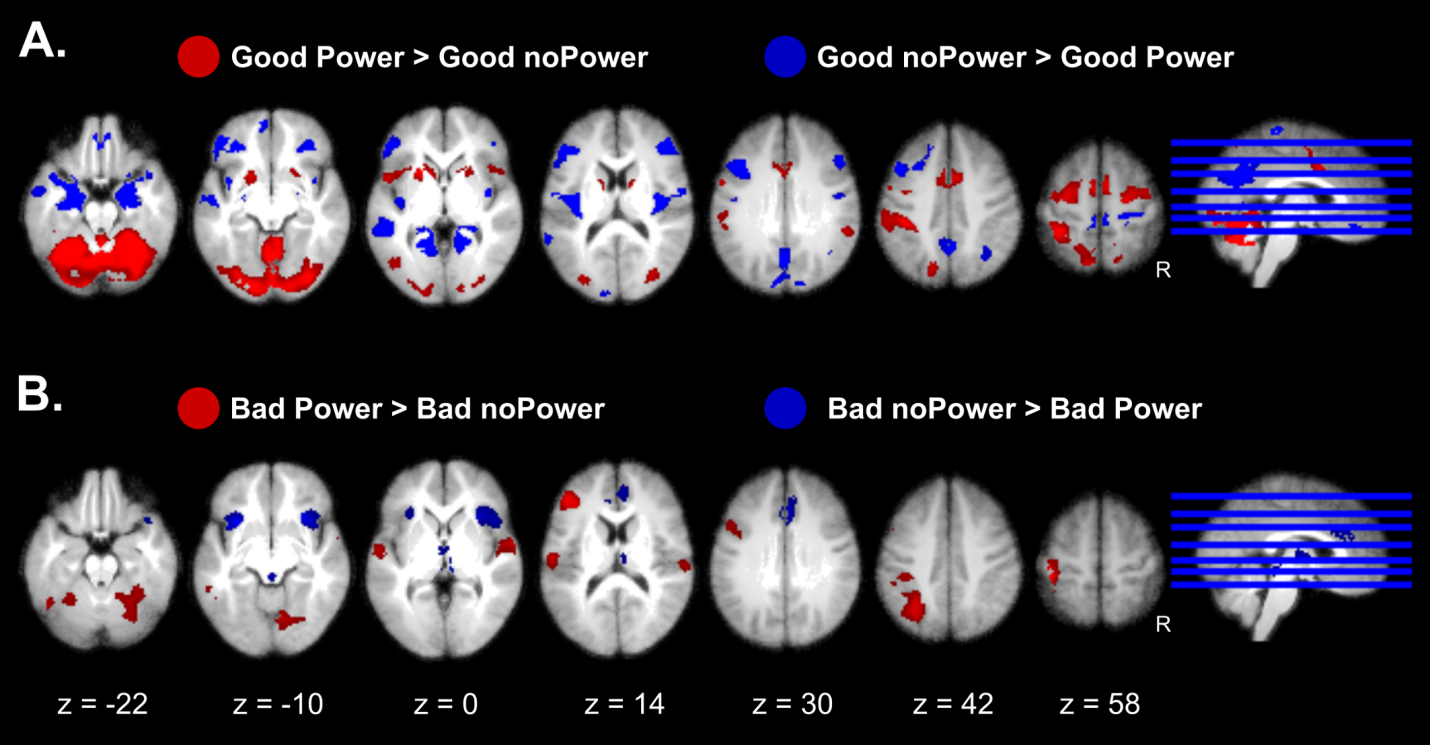
 S1 **Fig E. (Related to Discussion) Effects of ‘Coping Potential’ are shown separately for (A) good and (B) bad monster conditions.** Effects are presented on axial slices of a mean image created by averaging the participants' normalized structural images, with a height threshold of *p* < 0.001 uncorrected and a cluster-level threshold of *p_FWE_* < 0.05. Individual beta maps used for this figure are available on Neurovault at https://identifiers.org/neurovault.collection: 8740.


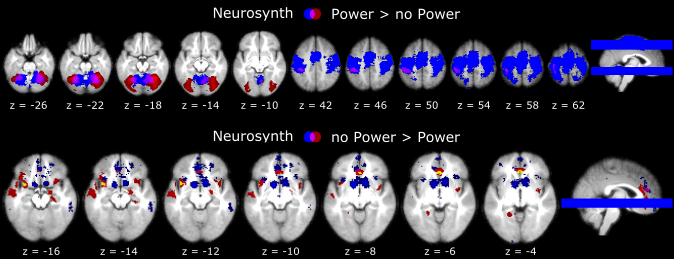


**S1 Fig. F. Overlap between Neurosynth reverse inference maps and Coping Potential contrasts.** To provide extra support for our interpretation of the appraisal component networks activated in our study, we built upon the Neurosynth database [Yarkoni, T., Poldrack, R. A., Nichols, T. E., Van Essen, D. C., Wager, T. D. (2011). Large-scale automated synthesis of human functional neuroimaging data. Nature methods, 8(8), 665. 10.1038/nmeth.1635], which enables data-driven reverse inference about potential cognitive functions or domains associated with distributed patterns of activation. We focused on topic-based data-driven reverse inference and used the latest version of the list of 200 topics provided by Neurosynth (v5-topics-200). (Upper panel) We selected the only topic (No 134, based on 1160 studies) that included all of the terms we used in our interpretation, namely: “action”, “motor”, “control” and “planning” (blue) and plotted it together with the contrast map for Power > No Power conditions in our study (red). As shown in the figure, areas related to higher coping potential in our study are included in the reverse inference map associated with this topic. (Lower panel) We repeated the same approach for the opposite contrast No Power > Power [corrected at p_FWE_ < 0.05 with (red) and without (yellow) applying a height threshold p_uncorr_ < 0.001] and searched for the topic containing the term “uncertainty” with associated terms that were closer to the context our task (No 186, based on 618 studies; in blue). The figure shows that the insula and ACC are constituent areas of the Neurosynth map attributed to uncertainty, overlapping with those modulated by lower coping potential in our study. We therefore feel confident that our short-hand description of this component network is valid and consistent with previous literature. Please note however that these comparisons do not imply that Coping Potential appraisals are equivalent to action control or uncertainty monitoring. Individual beta maps used for this figure are available on Neurovault at https://identifiers.org/neurovault.collection: 8740.


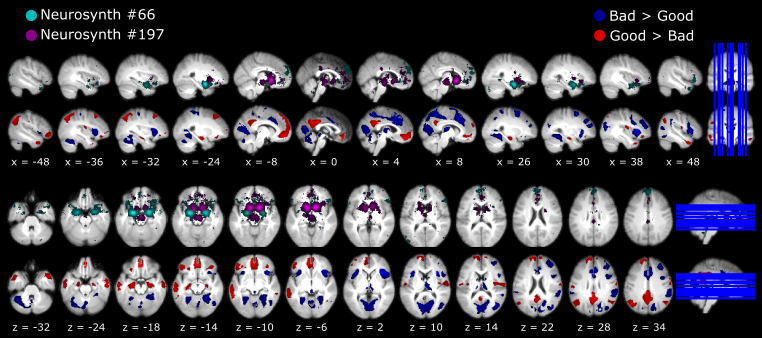


**S1 Fig G. Overlap between Neurosynth reverse inference maps and Goal Conduciveness contrasts.** To provide extra support for our interpretation of the appraisal component networks, we built upon the Neurosynth database (see figure S6 for details). For Goal Conduciveness we defined our topic search based on the following terms: “reward”, “punishment”, “valence” and “salience”. We found two topics, one containing the terms reward and punishment (no 197, based on 716 studies), and the other containing the remaining terms (No 66, based on 922 studies). Neurosynth reverse inference maps for topics 66 and 197 are depicted in cyan and violet, respectively, shown at the same sagittal (upper) and axial (lower) slices as the contrast maps for Good>Bad (red) and Bad>Good (blue). As can be seen, there is considerable overlap between our maps and the maps from Neurosynth in regions relevant to our interpretations, namely the amygdala, insula, vmPFC, PCC, thalamus, and ACC. Although these areas are not uniquely involved in reward and punishment/valence processing, these comparisons accord with our experimental manipulation of goal conduciveness through gains and losses and indicate that our short-hand description of this network dovetails neatly with previous literature. Please also note that this does not imply that Goal Conduciveness is equivalent to reward and punishment values. Individual beta maps used for this figure are available on Neurovault at https://identifiers.org/neurovault.collection: 8740.
