# Supplementary material for: Computational imaging during video game playing shows dynamic synchronization of cortical and subcortical networks of emotions
Source: PLoS Biol. 2020 Nov 12;18(11):e3000900. doi: 10.1371/journal.pbio.3000900 (PMC7685507; doi:10.1371/journal.pbio.3000900)
Supplement: Attachment — Submitted filename: PlosBio_ReviewersResponse_4submission.docx [file pbio.3000900.s007.docx]

We were grateful to read the positive comments from Reviewers and found their remarks very helpful and constructive. In this revision, we carefully addressed all questions that were raised. Below we provide a detailed answer to each and every of the reviewers’ comments.

REVIEWS:

***Reviewer #1:***

*The authors examined the human brain activity evoked while playing games and found several distinctive functional domains in cortical and subcortical areas. The approach is unique and powerful and can assess interactive and self-relevant cognitive components in a controlled manner.*

*My concern is about their claim regarding synchronization. The authors argued about the existence of recurrent and dynamic synchronization across functional components (Fig. 1). However, given the slowness of fMRI signals, the results cannot provide strong evidence regarding the underlying interactive process. Instead, a more straightforward interpretation is that each brain region is involved in multiple distributed cognitive processes, as reported in recent literature (e.g., Shine et al., 2019; Nakai and Nishimoto, 2020) without assuming recurrent synchronization. The authors should elaborate on potential alternative interpretations and clarify what the current results can and cannot resolve.*

We thank the reviewer for their comment. We recognize our wording might have inadequately underscored the recurrent dynamics of reciprocal interactions between component networks and synchronization, and now more cautiously articulate this point and focus on those aspects that were specifically examined by our fMRI approach. Nonetheless, the key implication of our study goes beyond the idea that brain regions are involved in multiple cognitive processes or overlapping networks, but additionally (and critically) demonstrates a temporally coordinated activation of regions mediating different emotion components. Our hypothesis and interpretation directly stem from the theoretical framework of the Component Process Model (CPM) which states that transient dynamic synchronization episodes are obtained through recurrent interactions between emotion components (characterized at the psychological / cognitive level rather than neural level in this framework). Here, we used the CPM to motivate and guide our experimental approach, with the aim of determining a neural signature of synchronization between networks at a coarse whole-brain system level through fMRI. But indeed, as noted by the Reviewer, our temporal resolution cannot reliably assess precise time-resolved recurrences in these neural interactions (we only assume they occur based on the CPM framework and look at their emergence at a more global scale in the fMRI time-course). Therefore, even though we do show synchronization between networks, it is true that fMRI resolution cannot test for recurrences at the brain level with high precision, and we now make sure to avoid any such misunderstanding by carefully rephrasing our text. Please note however that one of our two complementary analyses to tackle network synchronization used a computational model derived from CPM (Meuleman, 2015), which allowed us to derive a peripheral synchronization index and included recurrent feedback loops to estimate synchronization episodes (see also response to point 3 from Reviewer 2). Remarkably, results of this analysis neatly converged with the data-driven (brain-based) measure of synchronization. In the revised manuscript, we provide more information on parameters associated with synchronization and recurrence in our computational modelling with liquid state machines (ELSA model).

Importantly, despite being a slow measure of brain activity, fMRI time-course still allows testing for dynamic fluctuations and transient synchronizations between brain networks at a global mesoscale level relevant for characterizing emotional responses, and presumably resulting from reciprocal and/or recurrent interactions between component processes. In fact, instantaneous phase synchronization, the measure we used to define the synchronization index at the brain level, has been extensively used in prior fMRI studies investigating synchronization between different brain regions or brain networks, for example to study alteration in the dynamics functional connectivity due to neocortical focal epilepsy or during movie watching, and more generally to provide a characterization of the dynamics of interacting whole-brain systems(Glerean et al., 2012; Kitzbichler et al., 2009; Laird et al., 2001, 2002; Omidvarnia et al. 2016; Pedersen et al., 2017a, 2017b, 2018; Ponce-Alvarez et al., 2015). This assumption is also at the core of fMRI studies probing for dynamic functional connectivity among networks, such as the CAP methodology (Liu et al., 2013).

Finally, concerning the functional role of individual brain regions, we fully agree with the Reviewer it is likely that each region is involved in multiple distributed cognitive processes and these may reconfigure in different networks based on processing demands. This premise is not only a central starting point assumption but also an observation in our study (e.g. see lines 409-412, or 427-429). Yet, the way our synchronization maps were derived suggests that the regions involved were not simply co-activated across emotion components (i.e., these are not a mere “conjunction” of different “static” networks), but actually displayed a high degree of momentary synchronization in their activity time-course across different emotion components.

To make the distinction between transient and recurrent synchronization more explicit for the reader, and to clarify which parts of the CPM were addressed with our synchronization measures at the brain level and which were restricted to our computational model based on periphery synchronization, we introduced a short discussion in the limitations section of our manuscript (lines 503-510), and reviewed/adjusted our manuscript to ensure an appropriate and consistent use of the terminology (lines 360, lines 945-948). We also refrain from concluding about the recurrent aspects of synchronization processes.

*Optionally, it would be great if the authors could provide a discussion on whether there is a sort of emotion present in a game-playing AI agent (e.g., Mnih et al., 2015; Vinyals et al., 2019), which has internal states that correspond to the variables discussed in this manuscript.*

This is indeed a very interesting and thought-provoking question, touching upon the issue of whether computational models of affective processes might allow imbuing machines or robots with emotions (e.g. see Arbib et al., 2004). However, we feel like it falls outside the scope of this strictly empirical study, and addressing it would only add more complexity and speculations to an already complex dataset. We thus prefer not to include a discussion on this topic at this point.

**Reviewer #2:**

*In this paper, Leitão and colleagues describe research that explores how appraisal, motivational, expressive, and physiological components contribute to the emergence of emotion. The research uses an interesting combination of a novel experimental paradigm, fMRI, and computational approaches to show the neural correlates of these components during emotional experiences, and how they interact. This manuscript has multiple strengths: it focuses on a timely and under-studied problem, it is clearly theoretically motivated, it includes multiple measures of emotion in addition to fMRI, and the statistical procedures appear appropriate. However, the significance of the results are somewhat difficult to appreciate as interpretation of findings relies on reverse inference and there are a few critical methodological and conceptual issues, as detailed below.*

We would like to thank the reviewer for their encouraging comments and for having carefully revised our manuscript to clarify the interpretation of our findings. Specifically, we made sure to avoid reverse inferences, and rephrased our text accordingly in several places to avoid misinterpretations of our conclusions. Please note we do not infer any specific psychological mechanisms from our neural data (i.e., we do not seek to “explain” or “unpack” emotion components or appraisals in terms of particular information processing operations inferred from brain activity patterns, but instead compare brain networks identified here to those engaged by particular cognitive functions in strict reference to extant neuroscience literature.

First of all, concerning the risk of reverse inference, we underscore that apart from the appraisal components defined a priori by our experimental task design, brain maps for all other components resulted from using fMRI data to directly predict empirical measures of the corresponding behavioral, physiological, and expressive counterpart. For the appraisal component, we chose to select two major and well-established cognitive appraisal processes (Goal Conduciveness and Coping Potential) based on the CPM theoretical framework, and we manipulated these experimentally in order to delineate corresponding brain networks, which then served to compute synchronization with other networks. However, we do not use these networks to propose a psychological interpretation of appraisal processes. We return to this point in more detail below to answer to the corresponding reviewer’s major point No 2.

Second, our study does NOT claim that individual network mappings are uniquely linked to any given cognitive or affective process. Rather, our interpretations are meant to provide a descriptive account of the data in the context of the manipulated task demands and the measured behavioral/physiological responses. In fact, one fundamental premise of our study is that many previous fMRI emotion studies (as well as our own) reveal large-scale, *overlapping* activations resulting from different affective (and cognitive) processes. Critically, we argue such findings may be more readily compatible with componential theories of emotion, which have scarcely been explored with fMRI, unlike other theoretical models assuming categorical or orthogonal dimension. More specifically, based on appraisal theories, we aim at identifying different brain networks associated with different components in order to derive a measure of their synchronization. Moreover, while here we rely on a solid and previously validated theory based on CPM (Scherer, 2001), future work may extend our current approach to other appraisals and/or additional components. The significance of the current manuscript thus lies in demonstrating the feasibility of going beyond discrete and dimensional models and to focus on non-experiential aspects of emotion in a controlled and systematic manner, with self-relevant tasks, thereby promoting future emotion fMRI studies to pursue this important research direction that would allow tackling so far untested assumptions of emotion theories.

Major issues:

1. Does the experimental manipulation have external validity? It seems like the types of negative emotions the CPM tries to explain (e.g., responding to a dangerous, life-threatening event) are quite different than playing a video game in the fMRI environment. To what degree are the self-report and brain measures valid indicators of "real life" emotions?

Our experimental manipulations and the appraisal processes studied here (Goal Conduciveness and Coping Potential) were derived from a well-established model and solid evidence from past research in psychology (Scherer, 2001, Smith & Lazarus, 1993; Ortony et al., 1988; Roseman, 2001, Frijda, 1993). This is precisely why we chose the CPM framework for our work. In addition, the CPM (and other appraisal models of emotions) do not focus on negative emotions only, but in fact attempts to account for a wide range of emotions in general by identifying their relevant components (instead of emotion category-specific modules or dimensions). For example, a goal conducive event can be associated with pleasure or joy. The CPM also extends beyond life-threatening events (e.g., fear), related to simple goal frustration (e.g., irritation), losses (e.g., sadness), challenges (e.g., anger, pride), or norm-violations (e.g., disgust, guilt, shame; see Scherer, 2005).

In addition, by introducing video gaming with direct self-implication, our study aimed at higher ecological validity to study emotions compared to many other standard approaches using, for example, pictures of facial expressions. However, we do not claim this is the only useful approach. Further, we do recognize that video games alone might not allow the exploration of the full range and complexity of emotions experienced in “real life” situations (e.g. like grief), but we believe they still enable the experience of wide variety of emotions like joy/satisfaction/amusement or frustration and anxiety or regret in a powerful manner since they are directly self-relevant (unlike face pictures). Overall, in our opinion, video games represent the most valid choice for the current study in terms of multiple aspects:

1. **Practical feasibility**: Within the limitations of a scanner, participants cannot execute tasks that would require complicated actions and whole-body engagement. The game by contrast is simple to control and transfers some of that whole-body engagement to the game character, which can be as free as the game environment allows.
2. **Adherence to CPM assumptions**: The game allows a manipulation of the assumed emotion induction mechanism (in this case appraisal) by presenting systematically modified scenarios which, when appraised as intended, can trigger changes in the other emotion components assumed by the CPM: action tendencies, physiology, expression, and feeling.
3. **Emotional totality**: Because of the previous point, the induced emotions are expected to be more componential and more intense than classical induction paradigms, such as the viewing of pictures or videos, or free recall. While the latter type of task would otherwise also be fMRI-compatible, they are passive and do not manipulate appraisals nor engage all components of emotion.
4. **Literature precedents**: Other papers that investigated emotion processes outside the fMRI environment in the context of appraisal theory have successfully relied on video games (e.g., Kappas, 1999; van Reekum et al., 2004), sometimes using similar Pacman-type games (Lanctôt & Hess, 2007).

For all these reasons, we believe the choice of a video game is sufficiently ecological and powerful to induce emotions in an fMRI environment, without relying on passive stimulation and pre-defined emotion categories.

Finally, regarding the validity of our measurements to capture emotions in general, we have taken a comprehensive approach in our study. Although none of the individual measurements could be said to adequately capture or prove the occurrence of “objective emotions” when taken in isolation, we did apply a combination of criteria to define particular emotionally-relevant episodes, which is more than what most emotion studies in neuroscience typically rely on. Specifically, we observe (a) experimental conditions manipulated according to well-established appraisal criteria, (b) a post-hoc manipulation check indicating that participants indeed made the intended appraisals, (c) changes in action tendencies directly measured from behavioral performance in the video game task, and (d) a post-hoc manipulation check suggesting changes in qualitative/quantitative feelings measured through affective ratings along standard scales. Taken together, all of these aspects are strongly suggestive of emotions having been objectively triggered by the experiment. We now further emphasize these points in the revised manuscript (lines 393-398).

2. I*nterpretation of different brain systems largely relies on reverse inference, where the functional relevance of different brain regions is assumed rather than tested. Brain regions associated with increased coping potential were interpreted to reflect enhanced motor action control and planning during power. What are the relationships between brain activity associated with the pattern of brain activity observed with coping potential? Are they associated with measured differences in behavior in this way?*

We feel the Reviewer partly misinterpreted our conclusions and actually goes beyond what we intended to conclude. We therefore carefully rephrased relevant parts (e.g., pages 9-10) in the paper to avoid the impression that our results propose a particular function to particular brain regions or networks (see also our response to the main point above), and we better emphasize instead our main focus on the perspective of distinct but overlapping networks that relate to different emotion components and exhibit dynamic time-course and synchronized activity in emotional situations.

Concerning each of the networks modulated by specific appraisal conditions, our interpretation was not simply based on the activated areas but on empirical observations of participants’ gameplay, which are backed up by the action tendencies measures we report. Specifically, through the main effects of Coping Potential on the number of EatenCoins and other approach indices, we show a change of the participants’ focus from coins to monsters (and vice-versa), directly measured in behavior and reflecting different movement strategies within the maze. The most flagrant example of this was observable during the different Coping Potential levels of the “good monster” condition, where we show an increased amount of approaches to the monster in the Power condition and a decreased amount of eaten coins. Given that the monster is a moving target (as opposed to coins that are still) and goes in an unpredictable fashion 15% of the time in this condition, our findings imply that action planning is necessary to approach the monster and thus engaged by Coping Potential. However, this does not mean that we intended to conclude that Coping Potential is equivalent to planning or that brain areas modulated by the Power condition have a specific role in motor planning. As mentioned above, the interpretations we provide when reporting networks modulated by different appraisals are only meant to (1) offer a descriptive account of the data in the context of the manipulated task demands, and (2) confirm the validity of our approach by relating our fMRI results to current knowledge of brain systems or past studies. This is why we write, for instance, that brain networks activated by our manipulation of Coping Potential are consistent with previous evidence that similar areas are associated with motor and action planning functions, which is in turn consistent with objective task parameters. We made sure to clarify and unpack these points in the revised paper.

Nevertheless, given that such behaviors are not explicitly measured nor uniquely linked to fMRI activations, in order to provide extra support for our interpretation, we also build upon the Neurosynth database (Yarkoni et al., 2011), which enables data-driven reverse inference about potential cognitive functions or domains associated with distributed patterns of activation. In order to provide increased sensitivity and construct validity, instead of considering the individual features in isolation (where they can potentially have multiple interpretations; e.g., control could be equally associated with cognitive or motor control), we opted to focus on topic-based data-driven reverse inference (Poldrack et al., 2012). In particular, we used the latest version of the list of 200 topics provided by Neurosynth (v5-topics-200) and selected the only topic (No 134; based on 1160 studies) that included all of the terms we used in our interpretation, namely: “action”, “motor”, “control” and “planning”. The other features included in this topic were globally also consistent with our descriptive functional interpretation of the relevant network (complete list of topic features: motor, cortex, supplementary, sma, premotor, primary, sensorimotor, sensory, pre, network, control, somatosensory, cerebellum, task, hand, movement, planning, dorsal, cerebral, execution, passive, pattern, putamen, visuomotor, pmd, output, studied, play, action, bilaterally, movements, simple, voluntary, important, visually, involving, central, secondary, cerebellar, hemisphere). As shown in the figure below, the areas related to Coping Potential in our study are included in the reverse inference map associated with this topic. We also repeated the same approach for the opposite contrast (no Power > Power) and searched for the topic containing the term “uncertainty”. From the two identified topics (no 128 and 186), we selected the one whose terms were closer to the context our task (no 186, based on 618 studies, complete list: decision, making, choice, decisions, choices, uncertainty, outcomes, discounting, outcome, striatum, rewards, options, valuation, preferences, make, values, preference, probability, reward, subjective, individual, gains, economic, losses, loss, option, process, choose, aversion, gambling, insula, reputation, risky, selection, intertemporal, accumulation, people, prior, igt, choosing). Figure 1 (lower panel) shows that the insula and ACC are constituent areas of the Neurosynth map attributed to uncertainty.

We therefore feel confident that our short-hand description of this component network is valid and consistent with previous literature. Further, we carefully refrain from making reverse inferences on psychological underpinnings of appraisal components. Moreover, to back up our interpretation terminology, this new figure was also added in the supplementary materials.

Figure 1. (Upper panel) Overlap between Neurosynth reverse inference map (blue) for topic 139 (including motor, action, planning and control features) and the contrast map for Power > no Power conditions in our study. (Lower panel) Overlap between Neurosynth reverse inference map (blue) for topic 186 (including uncertainty features) and the contrast map for no Power > Power conditions in our study [corrected at p_FWE_ < 0.05 with (red) and without (yellow) applying a height threshold p_uncorr_ < 0.001].

*The authors also note that brain regions are modulated by Goal Conduciveness are reminiscent of networks often reported for positive/rewarding and negative/aversive stimuli. How well does brain activity predict variation in self-reported valence (e.g., for trials similar to those used to assay subjective feelings post-scanning)? Linking component processes to better-established behaviors could better corroborate the findings.*

The manipulation of Goal Conduciveness in our study was strongly based on manipulating reward (win points) and punishment (lose points), which we actually discuss as a point of consideration for future studies that may seek to separate Goal Conduciveness manipulations from this type of valuation processes. Additionally, despite being a manipulated parameter, we do acknowledge in the original discussion (lines 488-491) that this “*reminiscent pattern”* cannot solely be attributed to reward/punishment but, on the contrary, implies a more complex process (e.g. see also lines 409-413). In fact, this appraisal manipulation, due to the interactive nature of the task, did not allow isolating reward/punishment processing specifically. As mentioned above in our general response to the reviewer, this was also not our intent.

In fact, one of the goals of this study was to go beyond the dimensional and discrete approaches and purely subjective feeling ratings that are commonly employed in fMRI emotion research, but instead manipulate appraisal dimensions and focus on non-experiential emotion components. As mentioned above, while the appraisal of Goal Conduciveness is associated with a valence dimension, it cannot be completely explained by it. Moreover, in terms of dimensional approaches, specifically, we would also like to underscore that subjective valence ratings do not constitute a very robust behavioral measure either. In fact, there is no overarching agreement concerning the structure of subjective valence judgment (Barrett & Russell, 1998; Russell, 2017; Cacioppo et al., 2012; Larsen, 2016; Mattek et al., 2017), nor how it should be measured (e.g. the dimensional framework uses a bipolar scale with the assumption that as positivity increases, negativity decreases and vice-versa; yet, another bivariate framework uses two unipolar scales for positivity and negativity separately, with the assumption that positivity/negativity can decrease or increase without a corresponding change in negativity/positivity).

Thus, we believe that using fMRI data to predict subjective valence ratings, which were collected offline in a bundled fashion, using 5-point Likert scales (i.e., with a limited variability possibility) and screenshots to serve as post-hoc manipulation checks, would not only go against the fundamental idea that we wanted to address in this study, but also fail to constitute strong or informative evidence for interpreting our activations.

However, to underscore this point and provide consistency across appraisal manipulations, we also recurred to Neurosynth to back up our interpretations. For Goal Conduciveness we defined our topic search based on the following terms: “reward”, “punishment”, “valence” and “salience”. We found two topics, one containing the terms reward and punishment (no 197, 716 studies, complete list: reward, striatum, anticipation, ventral, monetary, rewards, motivation, loss, response, incentive, striatal, motivational, sensitivity, punishment, gain, outcomes, outcome, rewarding, money, delay, magnitude, mesolimbic, losses, gains, anticipatory, midbrain, salience, receipt, aversive, gambling, motivated, appetitive, rewarded, cues, feedback, incentives, dopaminergic, win, anticipated, avoidance), and the other containing the remaining terms (No 66, 922 studies, complete list: emotional, negative, amygdala, emotion, neutral, affective, valence, positive, arousal, emotionally, pictures, emotions, response, insula, limbic, affect, regulation, images, reactivity, ratings, valenced, suggest, impact, influence, viewed, arousing, context, salience, scenes, appraisal, presentation, viewing, intensity, structures, states, reactions, modulation, versus, dimensions, presented). As can be seen in the figure below, there is considerable overlap between our maps and the maps from Neurosynth in regions relevant to our interpretations, namely the amygdala, insula, vmPFC, PCC, thalamus, and ACC. Again, in the context of task demands and acknowledging that these areas are not specifically and solely attributed to reward and punishment/valence, we believe that our short-hand description is valid and consistent with previous literature.

Figure 2. Neurosynth reverse inference maps for topics 66 and 197 in cyan and violet respectively, shown at the same sagittal (upper) and axial (lower) slices as the contrast maps for Good>Bad (red) and Bad>Good (blue).

*3. Although some degree of neural synchronization takes place, it is not clear what drives this synchrony. Could it be explained by classic views of emotion where an appraisal of a particular event in the game triggers changes in autonomic physiology, expression, motivation, and feelings? Does the CPM make predictions about brain synchrony that differ from other theoretical views? How is recurrence needed to explain brain activity during the task? Do componential views offer insight here? In the absence of more direct comparisons between accounts, it is difficult to tell what we have learned from this interesting study.*

We felt there were two distinct angles to answering these questions, one purely theoretical, the other practical with respect to our data and modelling.

**Theoretical.** The CPM is currently—to our knowledge—the only theory that combines an appraisal theory of emotion causation, a componential view on emotion including the appraisals as one component, and a specific hypothesis connecting synchronization among emotion components to the resulting affective state. Competing theories may have a different causation hypothesis altogether (e.g., basic/discrete emotion theories), or not accept a componential definition of emotion (e.g., constructivist/dimensional theories), while other appraisal models lack the CPM’s synchronization hypothesis and instead focus on simpler (tree-structured) mappings of appraisal values onto different affective states (e.g., OCC model by Ortony et al., 1988; Roseman, 2001).

There has been some theory-driven work aiming to facilitate the comparison of appraisal models into a common language (Broekens et al., 2008), as well as data-driven work comparing a specific hypothesis common to several appraisal models (i.e., interactions among appraisal criteria; Meuleman & Scherer, 2012). However, the former are purely theory-driven efforts, while the latter necessitate the treatment of emotion data at a time-aggregated level for a valid comparison. For the current study, we designed a data-driven operationalization of the CPM that treated emotional data explicitly at a time-varying level, which other appraisal models (or theoretical integration efforts) do not address. There are alternative theories and models that do address affective states arising in context of (and sometimes from) time-dynamics (e.g., Cunningham et al., 2013; Joffily & Coricelli, 2013), including brain dynamics (Lewis, 2005), but these models do not sufficiently specify other components of emotion to facilitate comparison.

**Practical.** Although the fMRI methodology did not allow testing the recurrence dynamics explicitly at the brain-level—which is one of the features of CPM (see our response to reviewer 1 concerning this point)—our work incorporated this aspect at the psycho-physiological level in our fitted time-series model with liquid state machines (ELSA). The ELSA model gave us insight about **(a)** the extent to which feedback/recurrent influences among components was necessary to achieve accurate modelling, and **(b)** which component measures were contributing to the final measure of synchronization.

With regard to (a), the computational model contained an internal dynamic system (=liquid state machine) whose degree of *feedback recurrence* among emotion components was controlled by a specific parameter (lambda). Larger values for lambda mean more lagged feedback, while smaller values mean less lagged feedback, with 0 implying virtually no feedback recurrence. During model fitting, we optimized the value of this parameter with a rough cross-validation to arrive at a feedback length of ca. 5000 time steps. In other words, the data contained evidence that significant feedback was required for accurate modelling. This finding was confirmed with extra analyses—included now in the supplementary materials—that did not allow for feedback recurrence between components, and which produced significantly degraded model fits. In other words, the evidence for feedback contradicted the “classical” view of component interactions in appraisal theory as being a purely feedforward process (appraisal ⭢ motivation ⭢ physiology ⭢ expression ⭢ feeling; Moors, 2009).

With regard to (b), we found that the motivational measures were generally the best modelled by ELSA, with more non-zero parameters in their respective networks and better fit indices than the physiological measures, and expression measures, respectively. ELSA’s final overall measure of synchronization is essentially a combination of the component measures, weighted by how well they can be modelled. Hence, we can assume that this final measure of synchronization is largely driven by motivation, then physiology, and last expression. However, we caution to conclude that any component or process has a primary role in triggering synchronization and refrain from drawing conclusions regarding the causality that governs these component interactions. Please note that according to the theoretical assumptions of CPM, there is no unique sources of synchronization, hence this is an emergent state that may arise through different equilibrium levels among interactive components.

In sum, to our knowledge, our study is the first to define brain systems of emotion by 1) relating them to distinct components of emotion derived from a well-established theoretical model including appraisals and non-experiential features, 2) measuring the temporal dynamics of corresponding brain networks with transient moment of synchronization, and 3) combining computational modelling with brain data measures to identify neural substrates engaged by such synchronization. This approach is not incompatible with other views and approaches to emotion, particularly more descriptive accounts that link emotion to various manifestations and brain areas but (critically) do not incorporate synchrony as a core feature. As such, our work goes beyond previous fMRI work from both theoretical and methodological perspectives.

*Minor points.*

*1. The beginning of the introduction frames the paper around identifying essential neural circuits for emotion, but this is presumably not being tested with fMRI.*

We are not sure to have understood what the reviewer is refereeing to in this comment. While it is true that individual neurons and the circuits between them cannot be studied using fMRI at the micro level, large-scale neural “circuits” can be investigated using this technique at a macro level (i.e. networks), which is the level of analyses we are referring to in this study. Yet, to be sure to avoid any misunderstanding in this regard we replaced the term “circuits” by “networks” when referring to our own findings.

*2. The use of liquid states machines is interesting, but could perhaps be described more extensively for readers unfamiliar with the approach*

This is indeed an important feature of our computational modelling approach. Liquid state machines allow several time series (e.g., signals representing emotion measurements) to interact dynamically in a shared medium called the “liquid”, which is an artificial recurrent neural network with feedback connectivity in which activity gradually fades to 0 when no new input arrives. The latter is the so-called “fading memory” property of the model, and allows a time series that is inputted into the liquid to persist inside that liquid for a number of time steps (as a fading memory). Usually, the fading memory is also transformed with additional nonlinear functions (e.g., hyperbolic tangent). These liquid-transformed time series are then used in a second step as predictors in a conventional model, such as linear regression. The principle is that the liquid-transformed time series will capture more time-varying information than the original time series, and thus should result in more powerful predictors in subsequent regressions. The regression coefficients extracted from the second step of the analysis will encode transient attractor *trajectories* of the original time series. The liquid state machine itself only contains one permanent attractor *state*, its “dead state”, which it always reverts back to when no new input arrives. The ability of the model two encode these two types of attractors was a direct operationalization of the CPM model, which posits emotions as transient attractor trajectories—triggered by appraisal—permanently counteracted by a non-emotional baseline attractor state.

These aspects provide an important foundation for assessing the temporal dynamics of emotions as assumed in CPM and studied in the current work. We included a more detailed description of our liquid state machine approach in the corresponding methods section (lines 799-810).

3. *The discussion (and some of the introduction) suggests that most work on emotion does not focus on actual behavior. It is surprising that past work in this space is not cited or discussed, especially related work by Dean Mobbs using a similar manipulation with fMRI.*

It was not our intention to imply that behavioral measures had been completely overlooked in previous studies. Indeed, we do reference studies performed outside the scanner that not only used video games, but used them with the explicit purpose of appraisal manipulation (line 394). We had also referenced other similar fMRI studies in previous versions of the manuscript, which ended up being removed in the submitted version due to space constraints. These were now re-entered (line 389) as explicit exceptions to the generalized use static and passive manipulations. Note however that these approaches (e.g. by Dean Mobbs and colleagues) still often focused on emotion-specific (e.g. fear) modules rather than a multi-component view as we propose here.

We would like to thank the two Reviewers for the very insightful and constructive comments that helped us to clarify and strengthen our paper in many important ways.

**References**

Arbib, M. A., & Fellous, J. M. (2004). Emotions: from brain to robot. *Trends in cognitive sciences*, *8*(12), 554-561

Barrett, L. F., Russell, J. A., (1998). Independence and bipolarity in the structure of current affect. Journal of Personality and Social Psychology.; 74(4):967–984.

Broekens, J., Degroot, D., & Kosters, W. A. (2008). Formal models of appraisal: Theory, specification, and computational model. Cognitive Systems Research, 9(3), 173–197.

Cacioppo, J. T., Berntson, G. G., Norris, C. J., Gollan, J. K. (2012). The evaluative space model. In: Van Lange, P.Kruglanski, A., Higgins, ET., editors. Handbook of theories of social psychology. Vol. 1. Sage Press; Thousand Oaks, CA. p. 50-72.

Cunningham, W. A., Dunfield, K. A., & Stillman, P. E. (2013). Emotional states from affective dynamics. Emotion Review, 5(4), 344–355.

Frijda, N. H. (1993). The place of appraisal in emotion. Cognition and Emotion 7:357–87.

Glerean, E., Salmi, J., Lahnakoski, J.M., Jääskeläinen, I.P., Sams, M., (2012). Functional Magnetic Resonance Imaging Phase Synchronization as a Measure of Dynamic Functional Connectivity. Brain Connect. 2, 91–101.

Joffily, M., & Coricelli, G. (2013). Emotional valence and the free-energy principle. PLoS computational biology, 9(6).

Kappas, A. (1999). Don't Wait for the Monsters to Get You: A Video Game Task to Manipulate Appraisals in Real Time. Cognition & Emotion, 13(1), 119–124.

Kitzbichler M. G., Smith M. L., Christensen S. R., Bullmore E. (2009). Broadband Criticality of Human Brain Network Synchronization. PLoS Comput Biol 5(3): e1000314.

Laird A. R., Rogers B. P., Carew J. D., Arfanakis K., Moritz C. H., Meyerand M. E. (2002). Characterizing instantaneous phase relationships in whole-brain fMRI activation data. Hum Brain Mapp., 16(2), 71-80.

Laird A. R., Carew J. D., Meyerand M. E., Analysis of the instantaneous phase signal of a fMRI time series via the Hilbert transform, Conference Record of Thirty-Fifth Asilomar Conference on Signals, Systems and Computers (Cat.No.01CH37256), Pacific Grove, CA, USA, 2001, pp. 1677-1681 vol.2.

Lanctôt, N., & Hess, U. (2007). The timing of appraisals. Emotion, 7(1), 207.

Larsen, J. T., (2016). Holes in the case for mixed emotions. Emotion Review. 9 (2) pp: 118-123

Lewis, M. D. (2005). Bridging emotion theory and neurobiology through dynamic systems modeling. Behavioral and brain sciences, 28(2), 169–194.

Liu, X., Duyn, J. H. (2013). Time-varying functional network information extracted from brief instances of spontaneous brain activity. Proceedings of the National Academy of Sciences of the United States of America; 110(11):4392-7.

Mattek, A.M., Wolford, G., Whalen, P.J., (2017). A mathematical model captures the structure of subjective affect. Perspect Psychol Sci.; 12(3): 508–526.

Meuleman, B. (2015). Computational modeling of appraisal theory of emotion (Doctoral dissertation, University of Geneva).

Meuleman, B., & Scherer, K. R. (2013). Nonlinear appraisal modeling: An application of machine learning to the study of emotion production. IEEE Transactions on Affective Computing, 4(4), 398–411.

Moors, A. (2009). Theories of emotion causation: A review. Cognition and emotion, 23(4), 625–662.

Omidvarnia, A., Pedersen, M., Walz, J.M., Vaughan, D.N., Abbott, D.F., Jackson, G.D., 2016. Dynamic regional phase synchrony (DRePS): An Instantaneous Measure of Local fMRI Connectivity Within Spatially Clustered Brain Areas. Hum. Brain Mapp. 37, 1970–1985.

Ortony, A., Clore, G. L., & Collins, A. (1990). The cognitive structure of emotions. Cambridge university press.

Pedersen, M., Omidvarnia, A., Curwood, E.K., Walz, J.M., Rayner, G., Jackson, G.D., 2017a. The dynamics of functional connectivity in neocortical focal epilepsy. NeuroImage Clin. 15, 209–214.

Pedersen, M., Omidvarnia, A., Walz, J.M., Zalesky, A., Jackson, G.D., 2017b. Spontaneous brain network activity: Analysis of its temporal complexity. Netw. Neurosci. 1, 100–115.

Pedersen, M., Omidvarnia, J.M., Zalesky, A., Jackson, G.D., 2018. On the relationship between instantaneous phase synchrony and correlation-based sliding windows for time-resolved fMRI connectivity analysis. NeuroImage 181, 85-94

Poldrack, R. A., Mumford, J. A., Schonberg, T., Kalar, D., Barman, B., Yarkoni, T. (2012). Discovering relations between mind, brain, and mental disorders using topic mapping. PLoS computational biology, 8(10).

Ponce-Alvarez, A., Deco, G., Hagmann, P., Romani, G.L., Mantini, D., Corbetta, M., 2015. Resting-State Temporal Synchronization Networks Emerge from Connectivity Topology and Heterogeneity. PLoS Comput Biol 11, e1004100.

Roseman, I. J. (2001). A model of appraisal in the emotion system. Appraisal processes in emotion: Theory, methods, research, 68–91.

Russell, J. A., (2017). Mixed emotions viewed from the psychological constructionist perspective. Emotion Review. 9(2), pp: 111-117

Scherer, K. R. (2001). Appraisal considered as a process of multi-level sequential checking. In: Scherer KR, Schorr A, Johnstone T, editors. Appraisal processes in emotion: Theory, Methods, Research: Oxford University Press; p. 92-120.

Scherer, K. R. (2005). What are emotions? And how can they be measured?. Social science information, 44(4), 695–729.

Smith, C. A. & Lazarus, R. S. (1993). Appraisal components, core relational themes, and the emotions. Cognition and Emotion 7:233–69.

van Reekum, C., Johnstone, T., Banse, R., Etter, A., Wehrle, T., & Scherer, K. (2004). Psychophysiological responses to appraisal dimensions in a computer game. Cognition and emotion, 18(5), 663–688.

Yarkoni, T., Poldrack, R. A., Nichols, T. E., Van Essen, D. C., Wager, T. D. (2011). Large-scale automated synthesis of human functional neuroimaging data. Nature methods, 8(8), 665. 10.1038/nmeth.1635
