# Supplementary material for: Computational imaging during video game playing shows dynamic synchronization of cortical and subcortical networks of emotions
Source: PLoS Biol. 2020 Nov 12;18(11):e3000900. doi: 10.1371/journal.pbio.3000900 (PMC7685507; doi:10.1371/journal.pbio.3000900)
Supplement: Attachment — Submitted filename: PlosBio_Response2Reviewers_2ndRound_4submission.docx [file pbio.3000900.s008.docx]

**DETAILED RESPONSE TO THE REVIEWERS**

**Reviewer #1:**

*The authors provided some more details and discussions that support their original arguments regarding recurrent or feedback components. In particular, the additional Table S10 and the related discussion seem to be critical.*

*That being said, I am still not fully convinced, partly because they did not provide interpretable details of their methodology. The following information and control analysis will provide more concrete support:*

*(I) They should provide more methodological details on (A) feedback vs. (B) non-feedback models in Table S10. They could modify Figures 6/S2 to illustrate A and B. It is unclear what "lambda" indicates, as they do not show it in any equation, diagram, etc. It is also unclear what "rough cross-validation" means.*

*(II) Please provide the following negative and positive control simulation analyses:*

*(1) Prepare mock fMRI/behavior model signals with or without feedback components.*

*(2) Add correlated noise to (1).*

*(3) Apply temporal low-pass filtering to (2).*

*(4) Run the analysis pipeline (Figure S2?) to (3).*

*(5) Test analyses in Table S10.*

*If the simulation analyses above can detect the presence and absence of feedback components, that should be convincing evidence that their pipeline works to examine the difference, even using challenging fMRI data.*

We were glad the reviewer found additional information in our first revision useful. We now further revised our manuscript, expanding on methodological details.

In regards to (I), we would first like to emphasize that the technical details of the synchronization model are more exhaustively described in the theoretical chapter of the doctoral thesis (Meuleman, 2015) that originally developed it and is openly available online (https://doi.org/10.13097/archive-ouverte/unige:83638). Due to content and space constraints for the present paper, we were obliged to cut some corners on technical descriptions and present an abridged version. In reality, the length of the fading memory in a liquid state machine is determined both by **(a)** the lambda parameter and **(b)** a leaking rate parameter that allows fast or slow exponential decay of past states. In particular, lambda determines the amount of scaling applied to the liquid weight matrix W at each echo state, by forcing the maximum eigenvalue of the matrix W to be less than this value. This scaling takes place during the model’s initialization, when the liquid weight matrix W is constructed, so not technically part of any updating equation when the model learns from data. This is why lambda did not appear explicitly in any of the previous formulae. We have added a subscript to our equations, however, to make clear the dependency of the matrix W on this parameter.

In contrast, the leaking rate parameter has a more direct impact on fading memory length during model estimation, and it is optimized with cross-validation for best performance, as we did for our data. For the simulation without feedback that we conducted for the previous round of review corrections, lambda and the leaking rate were set to their “zero impact” values of 0 and 1, respectively. On a side note, it should be mentioned that the leaking rate was not present in Jaeger’s original paper on liquid state machines [Jaeger, H. (2001). The “echo state” approach to analysing and training recurrent neural networks. German National Research Center for Computer Science; Report No 148], nor in the earliest versions of our synchronization model. The addition of this parameter was an innovation of later authors [e.g., Holzmann, G. and Hauser, H. (2010). Echo state networks with filter neurons and a delay&sum readout. Neural Netw. 23(2):244-56]. Likewise, we apologize for the reductive description of our optimization process. The cross-validation procedure was actually a standard within-subject procedure based on leave-one-run-out approach, as described in the manuscript (lines 824-825) and now also in the supplementary materials section. The adjective “rough” in our original manuscript intended to mean that the optimization was not performed on a subject-by-subject basis with a systematic grid search of the possible control parameter space (i.e., all possible combinations of control parameter values). Instead, the parameters were determined based on pilot data and then a fixed set of parameters was applied to all subsequent participants. We acknowledge that a subject-wise optimization procedure that ran systematically through the parameter space would have been ideal, but was simply unfeasible from a practical/computational point of view. The “rough” adjective has been removed and more information on the optimization procedure has been included. We also extended the description of the extra analyses on recurrence effects in the supplementary materials, including additional discussion on the effect of the leaking rate parameter.

We opted not to follow the reviewer’s advice on adding information about recurrence to figures 6 and S2 nor running the analyses suggested in (II). In fact, based on the reviewer’s recommendation to change these figures (which are restricted to the methodology applied on the derivation of our *brain-based* synchronization index) and his/her suggestion to prepare fMRI mock signals to measure recurrence effects (and then use them to test analyses in table S10), we believe that there might be some misunderstanding on the reviewer’s side about the extra data we provided on recurrence and how these can be interpreted. Actually, the extra data we provided during the last revision round are ***restricted to psychophysiological data*** and to the ***peripheral synchronization index*** that is derived from the ELSA computational model and applied to test for brain network dynamics in our model-based analysis of the fMRI data. This cannot be transferred nor interpreted at a brain level, a point mentioned in our discussion of the previously submitted manuscript (lines 489-508):

“Additionally, this slow temporal resolution and our **brain-based synchronization approach are insufficient to uncover fast and recurrent interactions** among component networks during synchronization, as hypothesized by the CPM. Nonetheless, our computational model for the **peripheral synchronization index** did include recurrence as one of its parameter, allowing us refine our model-based analysis of network synchronization in ways explicitly taking recurrent effects into account (see Suppl. Material and Table S10). In any case, **neither the correlation of a model-based peripheral index nor an instantaneous phase synchronization approach could fully verify this hypothesis at the neuronal level using fMRI**. To address these limitations, future studies might employ other paradigms with different game events or other imaging analyses and methodologies with higher temporal resolution. Higher temporal resolution may also help shed light over causality factors hypothesized by the CPM, which could not be addressed here.”

The extra recurrence analyses introduced during the previous revision round were meant to provide some evidence of recurrent interaction between emotion components (at least at the psychophysiological level) as per reviewers’ requests, but we consider them to already fall outside the scope of the project. In fact, we prefer to focus the attention on aspects of synchronization we can study at the brain level (with the methodology used here), namely the occurrence of **transient** synchronization between components and their translation at the level of mesoscopic **brain networks** identified with fMRI. Moreover, the purely mathematical evidence that liquid state machines are capable of modelling delay and feedback effects in time series was already provided in its original papers (Jaeger, 2001; e.g., Glass-Mackey delay differential equation data) and a feedback simulation by Meuleman (2005) with artificial emotion data that contained feedback effects or no feedback effects.

We thank again the reviewer for his/her careful reading and thorough assessment of our paper which greatly helped us clarify the theoretical bases of our computational model as applied here to fMRI analysis. We feel the paper is now much stronger.

***Reviewer #2:***

*The authors have done an excellent job of addressing all points raised during review. This work is exceptional for its combination of theory, unique experimental design, and computational approaches. I hope it inspires similar research in this area.*

We are pleased to have addressed all the reviewer’s concerns and suggestions and are very grateful for this encouraging and positive feedback.
