# Supplementary material for: Computational imaging during video game playing shows dynamic synchronization of cortical and subcortical networks of emotions
Source: PLoS Biol. 2020 Nov 12;18(11):e3000900. doi: 10.1371/journal.pbio.3000900 (PMC7685507; doi:10.1371/journal.pbio.3000900)
Supplement: Attachment — Submitted filename: responseReviews_filler.docx [file pbio.3000900.s009.docx]

This was a final submission after editorial acceptance of the paper and with no extra reviews.
